# Cisplatin bioconjugated enzymatic GNPs amplify the effect of cisplatin with acquiescence

**DOI:** 10.1038/s41598-019-50215-y

**Published:** 2019-09-25

**Authors:** Sana Iram, Manaal Zahera, Iram Wahid, Abu Baker, Mohammad Raish, Altaf Khan, Naushad Ali, Saheem Ahmad, Mohd Sajid Khan

**Affiliations:** 1 Nanomedicine & Nanobiotechnology Lab, Department of Biosciences, Integral University, Lucknow, India; 2 Department Pharmaceutics, College of Pharmacy, King Saud University, Riyadh, 11451 Saudi Arabia; 3 Department Pharmacology and Toxicology, College of Pharmacy, King Saud University, Riyadh, 11451 Saudi Arabia; 4 Quality Assurance Unit, College of Pharmacy, King Saud University, Riyadh, 11451 Saudi Arabia

**Keywords:** Nanoparticles, Lung cancer

## Abstract

Enzymatic gold nanoparticles (B-GNPs) have been synthesized using a natural anticancer agent bromelain (a cysteine protease) and these nanoparticles were used to bioconjugate Cisplatin (highly effective against osteosarcoma and lung cancer). Cisplatin bioconjugated bromelain encapsulated gold nanoparticles (B-C-GNPs) were found profoundly potent against same cancers at much lower concentration with minimum side effects due to the synergistic effect of bromelain. The B-C-GNPs have been observed to inhibit the proliferation of osteosarcoma cell lines Saos-2 and MG-63 with IC_50_ estimation of 4.51 µg/ml and 3.21 µg/ml, respectively, and against small lung cancer cell line A-549 with IC_50_ 2.5 µg/ml which is lower than IC_50_ of cisplatin against same cell lines. The B-GNPs/B-C-GNPs were characterized by TEM, UV-Visible spectroscopy, Zeta potential and DLS to confirm the production, purity, crystalline nature, stability of nanoemulsion, size and shape distribution. The change in 2D and 3D conformation of bromelain after encapsulation was studied by Circular Dichroism and Fluorometry, respectively. It was found that after encapsulation, a 19.4% loss in secondary structure was observed, but tertiary structure was not altered significantly and this loss improved the anticancer activity. The confirmation of bioconjugation of cisplatin with B-GNPs was done by UV-Visible spectroscopy, TEM, FTIR, 2D ^1^H NMR DOSY and ICP-MS. Further, it was found that almost ~4 cisplatin molecules bound with each B-GNPs nanoparticle.

## Introduction

In both industrialized and developing countries, there is an increase in the number of reported cases of cancer. The report issued by the World Health Organization says that the number of new cases of cancer is over 10 million each year and annual deaths caused by cancer are over 6 million, whereas recent world statistics indicate that the number of new cancer cases will be more than 15 million in 2020^1^. Osteosarcoma is amongst the most common primary malignant tumor in children and adolescents. It is the eighth most common cancer in children^2^. The major characteristic of osteosarcoma is an unregulated proliferation of osteoid producing mysenchymal cells^3^. Recently, neoadjuvant and adjuvant chemotherapy along with surgery has increased the survival rate by 5 years for localized disease by more than 60% as compared to surgery alone^4,5^. However, the 5-year survival rate is below 30% in patients with metastasis continued to have poor prognosis^6^. The effective chemotherapy regimens currently utilized against osteosarcoma also have damaging effects on normal cells which result in acute life threatening complications. Currently, in chemotherapeutic treatment of osteosarcoma combinations of high doses of cisplatin^7^, with methotrexate^8,9^, ifosamide and doxorubicin^10^, have shown a significant improvement in the survival rate. However, there are various side effects associated with these anticancer drugs due to nonspecific uptake, such as necessitating the use of high dosages, poor blood supply in the case of osteosarcoma^11^, secondary malignancies and drug resistance phenotype^12^. Lung cancer is a leading cause of cancer related mortality and accounts for 23% deaths worldwide, which is higher than deaths related to colon, breast and prostate cancer combined together^13^. The reason behind the lethal effect of lung cancer is also a lack of effective chemotherapeutic strategies and diagnostic procedures to diagnose it at an early stage. Cisplatin drug is one of the highly effective drug against cancers, including lung cancer and osteosarcoma, which is also easily available these days, but due to its various side effects its application become limited^14,15^. Cisplatin/cddp is a platinum based chemotherapy drug which forms platinum complexes in cells, eventually, these complexes bind and result in cross linking of DNA finally triggering apoptosis. Its side effects include diarrhoea, loss of appetite, nausea, vomiting, and loss of taste may occur. Nausea and vomiting can be quite persistent and severe. Therefore, new approaches are needed since tumors are believed to reoccur in a short time after the first chemotherapy regimen^16^ and these chemotherapy drugs have multiple severe side effects on the body. It is necessary that new approaches such as to destroy at the level of individual cancer cells so that the spreading and progression of cancer cells will be reduced in the body. Recently, engineered nanoparticles become a significant tool in the treatment of cancer. They have the ability to either enhance the delivery of drugs, or uptake by target cells and/or reduce the toxic effect of free drug to other organs. Gold has been considered to have valuable properties for centuries^17^. Gold nanoparticles have attracted significant attention in targeted drug delivery^18–20^, diagnosis of disease^21,22^ and monitoring surgical procedures^23–25^ due to their unique combination of physical, chemical, electronic and optical properties. In the field of nanomedicine targeted drug delivery has been the main goal to achieve and gold nanoparticles playing a very significant role to achieve this goal as they can be easily synthesized, functionalized and they are also biocompatible. Due to the expansive surface to volume proportion of gold nanoparticles, an adequate number of drug molecules can be delivered by these particles^26^. Gold nanoparticles owing to their large size, prefentially accumulate at the tumor sites and in inflamed tissues. It is due to the characteristically defective architecture of the blood vessels that provide nutrients and oxygen to these tissues^27,28^. Once these nanoparticles squeeze out through these large vascular pores and into the tumor site, they remain stuck due to characteristically their low diffusivity and diminished lymphatic drainage. Developments in the field of nanotechnology are quite vigorous from the past few years and one of its important applications is targeted drug delivery. This can offer novel ways to deliver anticancer drugs to individual cancer cells and also capable in detection of cancer^29^. From the past ten years, the increase of nanomaterials in targeted drug delivery to increase the efficacy of treatment has been widely considered as a potential strategy. There have been various nanomaterials such as nanorods, liposomes, polymers etc. used for this purpose. In recent years, for the treatment of osteosarcoma different nanomaterials conjugated with the chemotherapy drugs such as LDH conjugated with methotrexate^30^, polymerized liposome nanoparticles conjugated with doxorubicin^31^ and calcium phosphate and lipid modified dextran nanoparticles conjugated with cisplatin and doxorubicin respectively^32,33^, have been used which proved the increase in efficacy of the drugs. Various chemical and physical methods have been adopted to synthesize different nanoparticles which can be utilized in targeted drug delivery systems^34^. The biological methods used for synthesis of nanoparticles have various advantages such as they are cheap, simple and yield nanoparticles at physiological pH and room temperature. Nanoparticles syntheses by biological route have high stability, monodispersity and can be obtained in large quantities^35–37^. Enzyme mediated synthesis of gold nanoparticles opened new directions in the field of combinatorial treatment against cancer. Bromelain^38,39^, Nitrate reductase^40^ and Trypsin^41^ mediated synthesized GNPs were discovered to be very convincing against cancer. Bromelain encapsulated GNPs were effectively used to bioconjugate several drugs against various diseases in site specific delivery in order to enhance the adequacy of medications.

Eventually, we synthesized monodispersed gold nanoparticles using pineapple based cysteine protease, bromelain which was utilized as a reducing and also as a surface functionalizing agent. In this study, cisplatin drug was used to conjugate with gold nanoparticles in order to deliver it specifically on the site directed delivery with great patient compliance. The characterization of B-GNPs and B-C-GNPs were done by UV-VIS spectroscopy, TEM, DLS and Zeta potential. Standard method of UV-Visible spectroscopy was used to estimate drug loading efficiency. The binding of cisplatin with B-GNPs was confirmed by FTIR and NMR spectroscopy. Furthermore, the cytotoxic effect of B-GNPs and B-C-GNPs were checked on cell lines Saos-2, MG-63 (osteosarcoma cells) and A549 (lung carcinoma cells).

## Experimental Section

### Materials

All the solvents, proteins and synthetic compounds were of analytical grade and were utilized as gotten from Merck and Sigma-Aldrich (St. Louis, MO, USA).

### Synthesis of gold nanoparticles

*In vitro* production of B-GNPs was done by incubating 1 mM H[AuCl4] (prepared in 50 mM Phosphate buffer) and 1 mg/ml bromelain in a reaction mixture of 3 ml. This reaction was carried out for 48 hrs at 40 °C temperature. A different reaction was performed without bromelain was utilized as a control. A sample was expelled several times after definite time period and dissected in UV-Vis spectroscopy to affirm the development of nanoparticles. On conclusion of the reaction, the unbound bromelain was expelled by using 50% v/v of ethanol followed by centrifugation (30,000 g, 30 min.), rinsed two times with milli Q water and utilized for further characterization.

### Study of conformational changes in bromelain by circular dichroism

Far-UV Circular Dichroism (Far-UV CD) estimates the change in configuration of natural macromolecules, including proteins. The variation in the structure of bromelain because of encapsulation over the surface of gold nanoparticles is estimated in expressions of CD mdeg. Far-UV CD estimations were done through Jasco spectropolarimeter demonstrate J-815 outfitted by way of a microcomputer. The machine was aligned with D-10-camphorsulfonic acid. The CD estimations were done at 37 °C with a thermostatically restricted cell holder joined to Neslab’s RTE 110 water bath through a temperature exactness of ± 0.1 °C. Native and encapsulated bromelain samples were put in 1 mm lane cuvette and spectra were captured in the wavelength ranging from 200 to 250 nm^42^. Every spectrum was the mean of 3 scans and was recorded at an interval of 1 nm wavelength. CD spectra were recorded at 20 nm/min as scan speed with 1 s as the response time. The enzyme concentration in these tests was 0.33 mg/ml.

### Fluorescence studies

Bis-ANS (Invitrogen, Eugene, OR) was dissolved at 0.1% w/v in Na-K phosphate buffer (8 mM Na_2_HPO_4_, 2 mM KH_2_PO_4_, 140 mM NaCl, pH 7.2). This solution was equilibrated at 4 °C for 3 days before it was used in experiments. The concentration of protein was taken 0.33 mg/ml. Fluorescence spectra were recorded with Carry Eclipse win FLR software of Agile Carry Eclipse Flourescence Spectrophotometer – MY16040008 at room temperature using a 10 mm path length cuvette. The appropriate blanks, run under the same conditions, were subtracted from the sample spectra. The protein solution was excited at 375 nm and the emission will be documented in the range of wavelengths 400–675 nm. Both the excitation and emission voltage were set at 700 V & slit width is 10 nm. The excitation & emission spectra were smoothed with Savitzky-Golay smoothing factor 10. Record.

### Bioconjugation of B-GNPs with anticancerous cisplatin drug

*In vitro* synthesized B-GNPs were bioconjugated to anticancerous drug cisplatin. The free amino moieties of cisplatin bind through the free carboxylate cluster available on bromelain by employing the activator 1-Ethyl-3-(3-dimethyl) carbodiimide (EDC)^43,44^. The 5 ml reaction mixture containing 50 mM HEPES buffer (pH- 6.0), 250 µg Cisplatin drug and 1 mM of B-GNPs with 5 mM EDC, in aliquots was used. The reaction was performed at 30 °C for 3 hours.

### Characterization of B-GNPs and B-C-GNPs

For characterization, UV-Vis spectrophotometry measurements were performed on a Shimadzu dual-beam spectrophotometer (model UV-1601 PC) in the wavelength scope of 220–800 nm in a quartz cuvette of 1 cm path length. The size and morphology of B-GNPs and B-C-GNPs were examined using a Tecnai^TM^ G2 Spirit BioTWIN FEI company Transmission Electron Microscope (TEM) operated at an accelerating voltage of 80 kV. TEM tests were set up by storing a bead (1 μL) of the produced B-GNPs and B-C-GNPs onto a carbon-covered copper plate and dried overnight. At that point, the samples were put in a vacuum chamber (~4 × 10−3 Torr) for 12 hrs. The estimation of sizes of the B-GNPs and B-C-GNPs were performed manually from the TEM images using Gatan digital micrograph. The mean particle size of B-GNPs and B-C-GNPs were estimated with a Dynamic Light Scattering (DLS) particle size analyzer (Zetasizer Nano-ZS, Model ZEN3600, Malvern Instrument Ltd, Malvern, UK). The powder of the sample was diluted to a concentration of 0.5% (Wt/v) in deionized water and sonicated for 1 min prior to estimation. The sample was taken in a DTS0112-low volume dispensable measuring cuvette of 1.5 ml. Mean particle dimension estimate was the mean of triplicate estimations for a solitary sample. Zeta potential was likewise estimated utilizing a Zetasizer Nano-ZS, Model ZEN3600 (Malvern Instrument Ltd, Malvern, UK).

FTIR confirms the secondary structure of native bromelain and bromelain attached to the surface of GNPs as a capping agent. Also, it confirms the binding of cisplatin drug with capped bromelain at the surface of GNPs. A film was prepared by placing a drop of the B-GNPs/B-C-GNPs solutions on a Si (111) substrate and vanishing of water was carried out by delicate warming and FTIR spectra of the pictures were confirmed on a Shimadzu FTIR-8201 PC apparatus worked in the disperse reflectance style at a declaration of 4 cm^−1^. To get great signal-to-noise proportions, 256 outputs of the bioconjugate film were taken in the range 400–4000 cm^−1^.

### Nuclear magnetic resonance (NMR)

The confirmation of binding of cisplatin drug with B-GNPs was also studied, for the very first time, through 2D DOSY ^1^HNMR. For the analysis, all the spectra were gathered on a Bruker 800-MHz NMR spectrometer outfitted with a triple-resonance TCI cryogenic probe. For 2D DOSY ^1^HNMR, stimulated echo bipolar gradient pulse experiments were utilized with a pulse hindrance of 5 ms following every gradient, a pulse field gradient length of 2.2 ms and with 15 s relaxation decay^45^. Chemical shift (δ, ppm) and diffusion coefficient (m^2^ s^−1^) was plotted against log concentration (molar).

### Drug loading efficiency

The percentage loading of cisplatin was assessed by analyzing the change in the intensity of the wavelength at 300 nm (λ300 nm) in the absorption spectra of the supernatant before and after the bioconjugation reaction. The change in intensity is dictated by substit\uting the estimations of A and B in Eq. (1). The absorbance was specifically estimated at 300 nm, the trademark OD of Cisplatin^46^.

1 $$Percent\,Loading\,of\,Cisplatin\,on\,B-GNPs=[(A-B)\,\ast \,\frac{100}{A}$$

where A- the absorbance of 250 µg cisplatin, B - the absorbance of unused cisplatin (in supernatant after bioconjugation of Cisplatin with B-GNPs), and the optical density (O.D.) of both A and B were taken at 300 nm^47,48^.

Drug loading efficiency of cisplatin was likewise computed by utilizing UV-Vis spectroscopy at 300 nm by calibrating standard graph of cisplatin. The standard graph was drawn by increasing concentration of cisplatin from 50 µg to 250 µg.

$$Percent\,Bioconjugation=\frac{Amount\,of\,drug\,bioconjugated}{Total\,drug\,added}\,\ast \,100$$

### ICP-MS analysis

The concentrations of Au and Pt were determined by ICP-MS (Thermo Scientific ELEMENT XR, a high performance, double focusing magnetic sector field was used to perform the experiment in IIT, Bombay) which was further used to calculate number of cisplatin molecules bound with single AuNPs.

### Anticancer studies of cisplatin conjugated Au-bromelain nanoparticles (*In vitro*) Cell culture

Human lung cancer cell line (A549) and human osteosarcoma cell lines (Saos-2 and MG-63) were gotten from National Center for Cell Science (NCCS), Pune, India. Saos-2, MG-63, and A549 cells were developed as a monolayer in Mac Coy’s 5 A, MEM and DMEM F-12 medium, respectively,supplemented with 10% fetal bovine serum and 1% antibiotic containing 10,000 units of penicillin, 10 mg streptomycin and 25 μg amphotericin B in a humidified climate containing 5% CO_2_ incubator at 37 °C. Stocks were kept up in 25 cm^2^ tissue culture flask.

### Study of synergistic effects of B-GNPs and cisplatin drug

Chou and Talalay explained the “Combination Index” in order to calculate the combined effect of two different drugs whose responses are linearly related to the dose itself. In the study, combined doses (d_1_ units of one drug and d_2_ units of second drug) of two different drugs were mixed to get 50% effect. The combined effect can be explained by calculating:

$$\begin{array}{rcl}CIA & = & \frac{{d}_{1}}{ED50{}_{1}\,}+\frac{{d}_{2}}{ED{50}_{2}}\\  & = & \{\frac{P}{(P+Q)ED{50}_{1}}+\frac{Q}{(P+Q)ED{50}_{2}}\}ED{50}_{c}\end{array}$$

$$where,\,{d}_{1}=(\frac{P}{P+Q})ED{50}_{c}$$

$${d}_{2}=(\frac{Q}{P+Q})ED{50}_{c}$$

ED50_c_ – combined effect of 50% inhibition; Q & P - amount of two different drugs used to get 50% effect. For combination to be synergistic CIA < 1, for antagonistic CIA > 1 and at CIA = 1, combination becomes additive^49^.

### Cell viability assay

MG-63, Saos-2 and A549 cells were plated in 96 well plates at a thickness of 1 × 10^4^ cells for each well and incubated for 24 hrs in a humidified 5% CO_2_ incubator at 37 °C. After 24 hrs, the cells were incubated with B-GNPs, B-C-GNPs and cisplatin at concentrations (20, 10, 5, 2.5, 1.25 µg/ml) in triplicates, and incubated for next 48 hrs. Following the incubation, the media was disposed of and 50 μl MTT [3-(4,5-dimethylthiazol-2-yl)− 2,5-diphenyl-tetrazolium bromide] (5 mg/ml in PBS), was supplemented to each well. The plate was incubated for 4 hrs in 5% CO_2_ incubator. The subsequent formazan crystals were solubilized in 150 μl DMSO (Dimethyl sulfoxide). The measurement of decreased MTT was completed by estimating the optical densities over a wavelength of 570 nm through mention channel of 655 nm utilizing an ELISA peruser [(Microplate Reader (BIORAD-680)]. Percentage inhibition of the cells was figured utilizing the formula $X=100-(Atest-Ablank)/(Acontrol-Ablank)\ast 100$; where X is percentage inhibition, Atest is the absorbance of the test sample, Ablank is the absorbance of blank and Acontrol is the absorbance of the control sample. The estimated IC_50_ was figured by fitting the information utilizing ORIGIN 6.1^50^.

### Measurement of cytomorphological changes in Saos-2, MG-63 and A549

MG-63, Saos-2 and A549 cells were pre-treated with various concentrations of B-GNPs, B-C-GNPs and unadulterated cisplatin and kept for 48 hrs at 37 °C in 5% CO_2_ environment. After the incubation of cells, the net morphological variations inside the cells were seen with the application of an inverted phase contrast microscope (Nikon ECLIPSE Ti-S, Nikon Corporation, Tokyo Japan).

### Study of changes in nuclear morphology

The apoptotic impact of B-GNPs, B-C-GNPs and cisplatin (pure) on MG-63, Saos-2 and A-549 cell lines was studied with the aid of fluorescent nuclear dye DAPI. The cells were seeded and treated like above. At that point the cells were rinsed with PBS and settled in 4% paraformaldehyde for 10 min. In this way the cells were permealized with permealizing buffer (3% paraformaldehyde and 0.5% Triton X-100) and stained by fluorescent dye DAPI. In the wake of staining, the pictures were obtained under a fluorescence microscope (Nikon ECLIPSE Ti-S, Japan). The cells with fragmented and condensed nuclei had been measured as apoptotic cells.

### Detection of reactive oxygen species

Generation of ROS in A-549, MG-63 and Saos-2 cells was estimated by incubating these cells with B-GNPs, B-C-GNPs and cisplatin (pure) was identified utilizing the Image-IT Green ROS Detection unit (Invitrogen). The observation take place on a fluorogenic marker, 5-(and-6)- carboxy-2′,7′-dichlorodihydrofluorescein diacetate (carboxy-H_2_DCFDA), for ROS in viable cells. The non-fluorescent carboxy-H_2_DCFDA pervades live cells and is deacetylated by non-specific intracellular esterases. Within the sight of non-specific ROS (created, especially throughout oxidative stress within the cells), the decreased fluorescein complex acquire oxidized and discharges brilliant green fluorescence. Cells (1 × 10^4^ for each well) were seeded in 96-well culture plates and permitted to follow for 24 h inside the CO_2_ hatchery at 37 °C. Cells were subsequently presented to various fixations (20, 10, 5, 2.5, 1.25 µg/ml) of B-GNPs, B-C-GNPs and cisplatin (pure) for 24 h. From there on, cells were incubated with H_2_DCFDA (10 mM) for 30 minutes at 37 °C. The response blend was suctioned and supplemented by 200 μL of phosphate buffer saline (PBS) in every well. The experiment was held in reserve on a shaker for next 10 minutes at room temperature. A reversed fluorescence magnifying lens (Nikon ECLIPSE Ti-S, Japan) was utilized to break down intracellular fluorescence of cells. Quantification of cellular fluorescence from fluorescence microscopy images was done by ImageJ software.

## Results and Discussions

### Results

**Figure 1:**
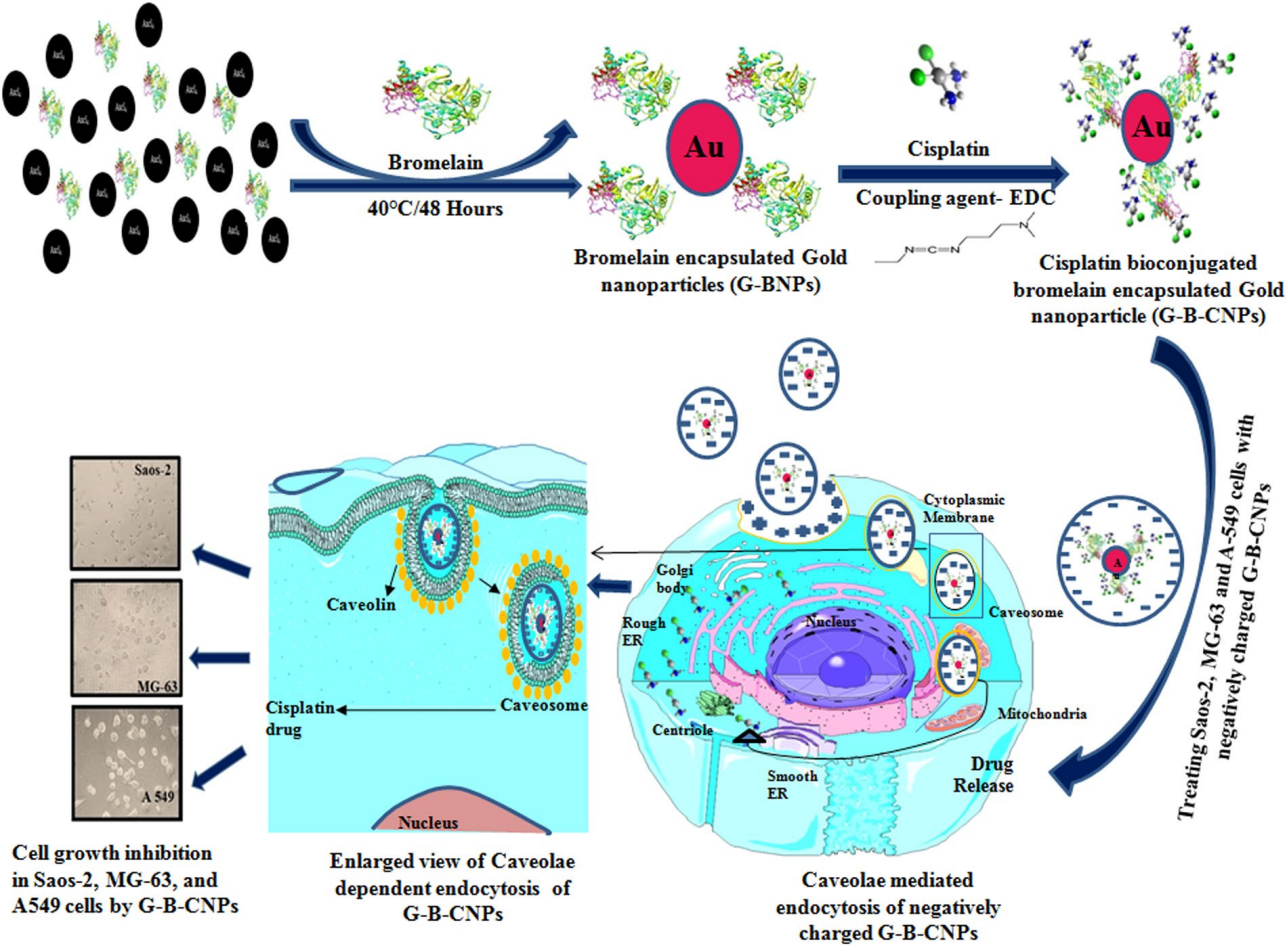
Schematic representation of bromelain assisted synthesis of gold nanoparticles, their conjugation with anticancerous drug cisplatin and caveolae dependent endocytosis to combat Saos-2, MG-63 bone cancer cells and A549 lung cancer cells.

In this study a novel method has been developed to synthesize size controlled bromelain mediated gold nanoparticles to use as a targeted drug delivery vehicle for cisplatin (Fig. 1). During encapsulation over the surface of gold nanoparticles bromelain undergo several 2D & 3D conformational changes and finally adopts its native structure with meager variations which do not alter the anticancer property of bromelain but increase the potency of cisplatin by acting synergistically against bone and lung cancer. The bioconjugation between –COOH group of bromelain and –NH_2_ group of cisplatin was performed by EDC and its confirmation was done by UV, TEM, FTIR and 1H^1^ NMR DOSY. The combo B-C-GNPs showed profoundly enhanced activity against MG-63, Saos-2 and A549 cell lines in comparison to the pure cisplatin drug alone because both bromelain and cisplatin work synergistically.

### Synthesis and characterization of B-GNPs

**Figure 2:**
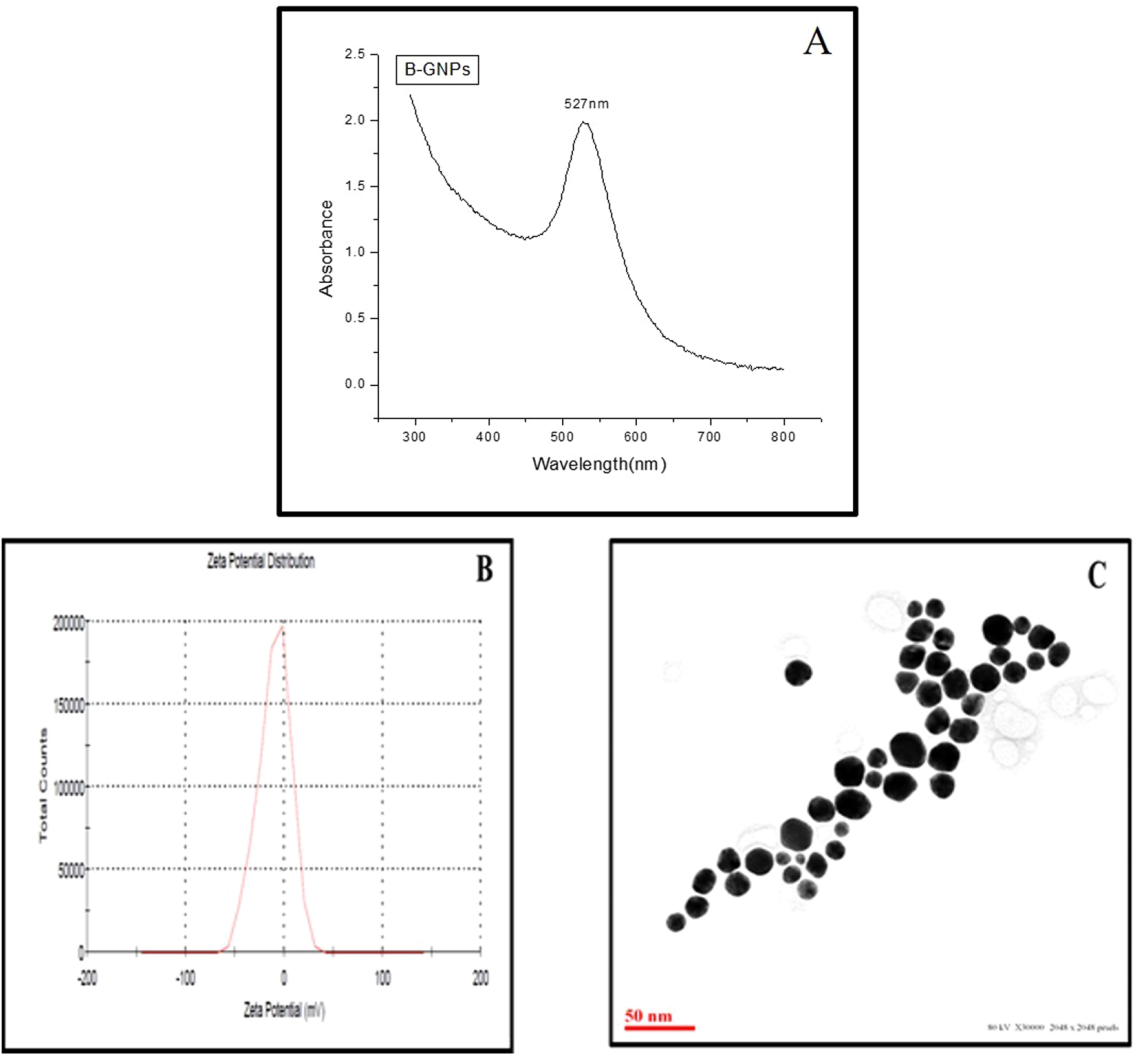
Characterization of B-GNPs under (A) UV-Visible spectra (B) Zeta potential (C) Transmission Electron Microscopy.

When bromelain, a cysteine protease, was incubated with 1 mM H[Aucl4], at temperature 40 °C for 48 hrs, resulted in the synthesis of gold nanoparticles with monodispersity and high stability. The synthesis of B-GNPs was confirmed by a gradual transform in color starting from light yellow to characteristic ruby red color subsequent to incubation with bromelain. The transformation in color is due to the surface plasmon resonance of B-GNPs. Further, it was confirmed by UV-Visible spectroscopy, and surface plasmon resonance absorption band appears at 527 nm corresponds to the plasmon band of B-GNPs (Fig. 2A)^48^. The synthesized B-GNPs are stabilized by bromelain, a strong capping agent with Cys, Asp and His in the active site which makes the nanoparticles stable by preventing them from aggregation. Most of the hydrophilic moieties, but not all such as hydroxyl/carboxylate/amino group present on protein will be involved in the bonding with nanoparticles during the process of capping. B-GNPs were found to be highly stable with negative charge and their zeta potential were found to be −10.1 mV (Fig. 2B). High resolution images of B-GNPs were acquired using TEM and their average sizes were estimated to be ~16 nm (Fig. 2C). Their shapes were found to be spherical and uniformly distributed under TEM.

### Study of conformational changes of bromelain during the process of capping fluorescence studies

**Figure 3:**
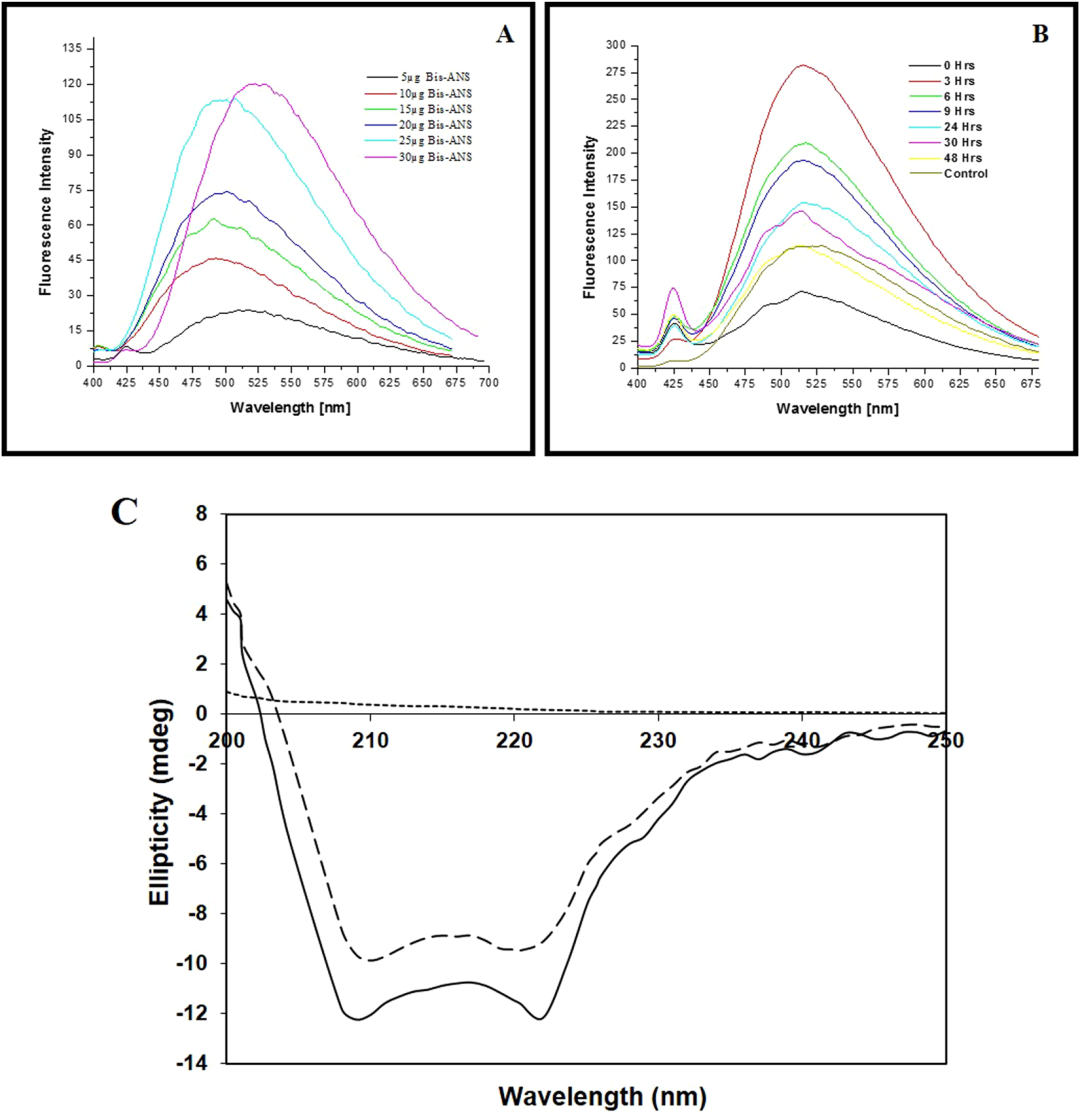
(**A**) Change of bis-ANS fluorescence intensity (fluorescence maximum, λ_517_ nm) in the B-GNPs reaction mixture at different incubation time. (**B**) Change of bis-ANS fluorescence intensity (fluorescence maximum, λ_517_ nm) at different concentration of bis-ANS against fixed concentration (0.33 mg/ml) of bromelain. (**C**) Far UV-CD spectra of pure Bromelain (―); Bromelain encapsulated gold nanoparticles (----); and chemically synthesized gold particle (…..).

In this study, bromelain mediated synthesized gold nanoparticles have been functionalized by bromelain itself. In this process of synthesis of gold nanoparticles, bromelain acted as a reducing as well as a capping agent. In order to investigate the change in the 3D conformation of bromelain during capping, bis-ANS dye has been used which interacts non-covalently to hydrophobic surfaces on proteins that are exposed during the process of capping. The polarity of the microenvironment of dye plays a critical role in its fluorescence. The complementary interactions of both apolar and polar groups of bis-ANS are the key feature used in the study of protein folding. Interestingly, it was observed that bis-ANS binding to proteins is dominated by hydrophobic interactions^51^. In general, native structure of a protein does have polar surface, exposing mostly hydrophilic moieties whereas hydrophobic moieties intent to bury inside. Before beginning the experiment, the optimum concentration of bis-ANS was standardized with the fixed concentration (0.33 mg/ml) of bromelain (Fig. 3A) and it was found to be 25 µg. Thus, in the beginning of the reaction (at 0 Hrs), very weak bis-ANS fluorescence was observed (Fig. 3B) because at this point of time gold was present in the form of Au^+^ ions and it interacts with bromelain through hydrophilic interactions (very few hydrophobic areas were present on bromelain). As the reaction proceeds (at 3 Hrs), maximum bis-ANS mediated fluorescence was observed because bromelain, by virtue of its protease nature, reduces Au^+^ to Au^0^ which enhanced hydrophobic interactions and caused unfolding of bromelain. With the progress of the reaction (till 48 Hrs) bis-ANS mediated fluorescence decreases with time due to involvement of hydrophobic sites of bromelain in the interaction with GNPs (Fig. 3B). Ultimately, after the complete synthesis of B-GNPs, bromelain adopts its native structure and shows no significant variation from normal.

### Circular dichroism

The structure of bromelain is comprised of an α helix (23%), β sheets (5% parallel and 18% antiparallel) and 18% turns with 35% residual part in various structures^52,53^. The sub-nuclear mass of bromelain is 23,800 Da with three disulfide bridges and a solitary free cysteine amino acid among cumulative 212 amino acids. Figure 3C shows the far-UV CD spectra of native (control) and bromelain gold nanoparticles (B-GNPs). There was an almost 19.4% loss in the secondary structure after formation of gold nanoparticles of bromelain as compared to that of control bromelain as observed at 208 nm. This can be expected to conformational change from the peptide native state in solution after binding to the nanoparticle surface, or potential energy exchange from the peptide to the metal nanoparticle. It is likely that the second probability may be the overwhelming dominant since the Bromelain peptide still held its primary helical highlights in light of the investigation of the CD spectra utilizing CD Pro software. Comparative wavelength shifts have likewise been seen upon protein adsorption to metallic surfaces^54^. Furthermore, upon modification induced in the secondary structure of protein as a consequence of nanoparticles formation, it leads to increase in mean residual ellipticity (MRE) value as compared to the control. It is interesting to note that although the secondary structure has loosed upon capping with gold nanoparticle, however the identity of secondary structure was retained as such as compared to the control. The structural retention is observed from the peaks at 208 and 222 nm of B-GNPs. This result signifies that structure has opened/unfolded upon modification induced by gold nanoparticles.

The UV CD spectra of stem bromelain with and without gold particles have been shown in Fig. 3C. The indigenous shape of bromelain is depicted by a characteristic peak around 280 nm likewise detailed somewhere else^53,55^. It can be seen from the figure that modified bromelain underwent insignificant variation in tertiary conformation after capping when compared with its control. It is fascinating to take note of that slight increment in ellipticity with the range looking like that of local bromelain arrangement, apparently because of the development of a relatively compact conformation.

The above information clearly showed that there was a change in 2D conformation of bromelain after capping over B-GNPs but 3D conformation didn’t alter significantly. This implies that slight loss in secondary structure does not lead to tertiary structure loss. Hence, it could be implicated that upon the formation of the B-GNPs, the functional properties as determined by the tertiary structure does not hamper, however, its secondary structure leads to the unfolding of the protein structure, which could cause its enhanced activity as supported by other experiments.

### Bioconjugation of anticancerous cisplatin drug with B-GNPs

**Figure 4:**
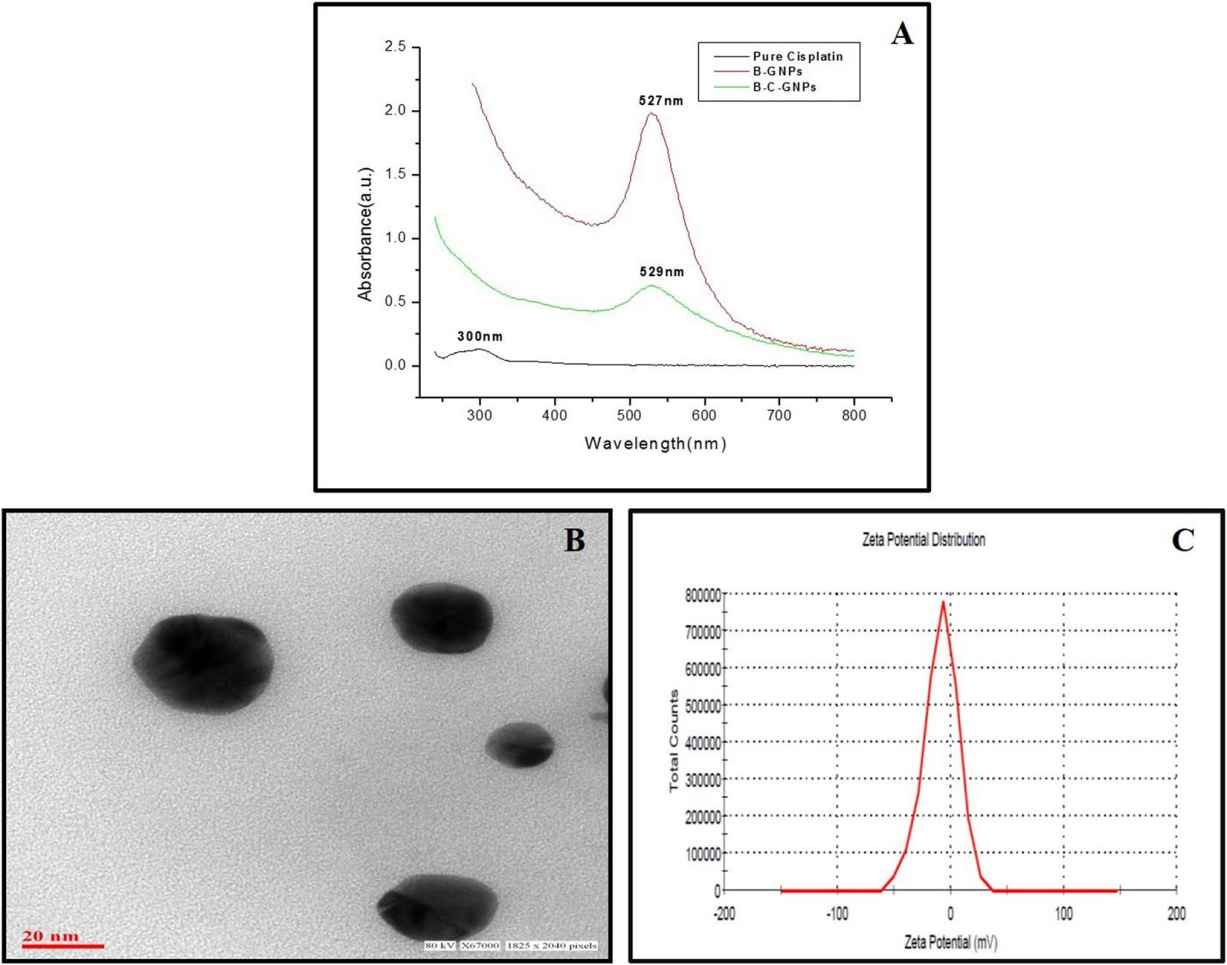
Characterization of B-C-GNPs under (**A**) UV-Visible spectra (**B**) Transmission Electron Microscopy (**C**) Zeta potential.

Thus synthesized B-GNPs were conjugated with anticancerous drug cisplatin using a carboxylate group of bromelain exposed over the surface of NPs with the amino group of cisplatin drug. Binding was achieved through the coordination bond between platinum atom and terminal carboxylate groups present on cisplatin and bromelain, respectively^56^. The surface plasmon resonance absorption spectra of cisplatin drug and B-C-GNPs are plotted in comparison to B-GNPs (Fig. 4A) and surface plasmon resonance absorption band of conjugated NPs was observed at 529 nm with decrease in intensity and a significant broadening compared to the absorption band for B-GNPs at 527 nm. The broadening of plasmon band and decrease in intensity is due to the attachment of the cisplatin drug to B-GNPs (Fig. 4A). With the attachment of any ligand on the surface of nanoparticles, the absorption intensity according to the surface plasmon resonance phenomenon substantially changes with a minor change in the full width half maximum of the absorption band^57^.

High resolution images of B-C-GNPs were acquired using TEM and their average sizes were estimated to be ~28 nm (Fig. 4B) which is bigger than B-GNPs with blur image. Their shapes were found to be spherical and uniformly distributed under TEM. Also, B-C-GNPs were found to be highly stable with negative charge and their zeta potential were found to be −8.97 mV (Fig. 4C) less than −10.1 mV for B-GNPs. There will be either red shift or blue shift in the UV- Visible spectra for conjugated NPs (B-C-GNPs) with a decrease in intensity (Fig. 4A) and TEM shows the increase in size with diminished sharpness in the picture (Fig. 4B) which are other proofs of bioconjugations^58^.

### FTIR spectroscopy of B-GNPs and B-C-GNPs

**Figure 5:**
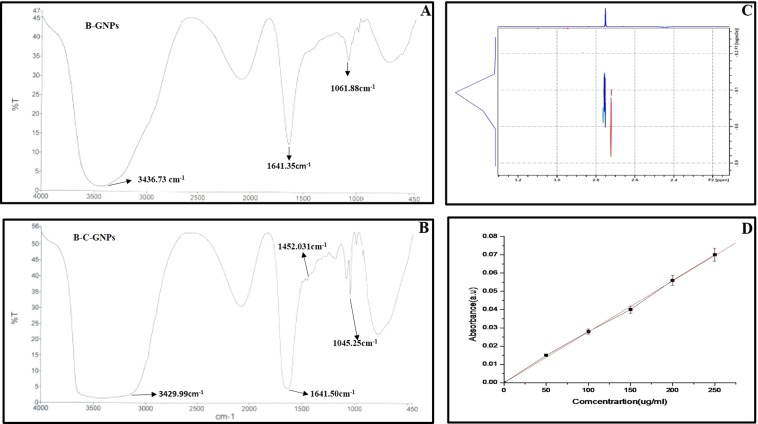
(**A**) FTIR spectra of B-GNPs (**B**) FTIR spectra of Cisplatin conjugated B-C-GNPs (**C**) 2D ^1^H NMR DOSY spectrum showing diffusion coefficients of B-GNPs and B-C-GNPs (**D**) UV-Visible spectra of pure cisplatin for drug loading efficiency.

The peptide bonds present in a protein are considered most significant and sensitive markers (as amide I and amide II) for secondary conformational changes under infrared area of the electromagnetic range^59^. The FTIR spectroscopy was used to detect the presence of capping agent at the surface of B-GNPs and their bioconjugation with cisplatin. A broadband at 3436.73 cm^−1^ (Fig. 5A) ensures the trapped water molecule in the B-GNPs films. These B-GNPs also show a characteristic peak of bromelain centered at 1641.35 cm^−1^ and 1453.03 cm^−1^. The bioconjugation of cisplatin with B-GNPs was confirmed by observing the broadening of peak at 3429.99 cm^−1^ which is the indicator of –NH stretching present in peptide bond with amide I and amide II at 1641.50 cm^−1^ ^60^ and 1452.03 cm^−1^, respectively. Further extra peak at 1045.25 cm^−1^ correspond to C-N of aliphatic amines stretch of peptide bond also confirms the bioconjugation (Fig. 5B).

### NMR spectroscopy

Further to describe the conjugation of B-C-GNPs, ^1^H DOSY NMR was performed to decide the modification in dissemination of the development of B-C-GNPs. With increase in size, the rate of diffusion (diffusion coefficient) diminishes which clearly indicates the formation of aggregates^61–63^. It was observed that there is a decrease in diffusion coefficient from 9.082 m^2^s^−1^ to 8.980 m^2^s^−1^ as drug conjugated to pure B-GNPs (Fig. 5C). This confirms the change in size of B-GNPs after attachment of drug molecule on the surface, as also shown by TEM.

### ICP-MS analysis

The concentration of Au and Pt were found to be 71.152 × 10^8^ and 16.18 × 10^8^ particles, respectively in the 5 ml reaction volume. Hence, it was calculated that almost ~4 cisplatin molecules bound with a single B-GNPs.

### Drug loading efficiency

The percentage loading of B-GNPs has been ascertained by utilizing Eq. (1) and observed to be ~79% showing a proficient attachment of cisplatin to B-GNPs. The estimations of A and B were acquired as 0.070 and 0.016 separately, and put in Eq. (1).

The quantitative estimation of cisplatin conjugated B-GNPs was also determined by another method of using UV-Vis spectroscopy (Fig. 5D). The absorbance of the pure cisplatin drug was observed at 300nm^47^ at five different concentrations (50, 100, 150, 200, 250 µg/ml). The amount of drug conjugated to B-GNPs was found to be ~79% revealing efficient binding of cisplatin with B-GNPs.

### Study of synergistic effects of B-GNPs and cisplatin drug

According Chou Talalay, Combination Index of B-GNPs and Cisplatin were calculated using 266.4 µg/ml concentration of B-C-GNPs and 38.5 µg/ml concentration of cisplatin in a definite combination to achieve 50% inhibition of MG-63, Saos-2 and A-549 cancer cells. The value of CIA against MG-63, Saos-2 and A-549 cells were found to be 0.442, 0.428 and 0.286, respectively which proved that B-GNPs and cisplatin work in a synergistic manner.

### An *in vitro* anticancer study of B-GNPs and cisplatin conjugated B-C-GNPs

**Figure 6:**
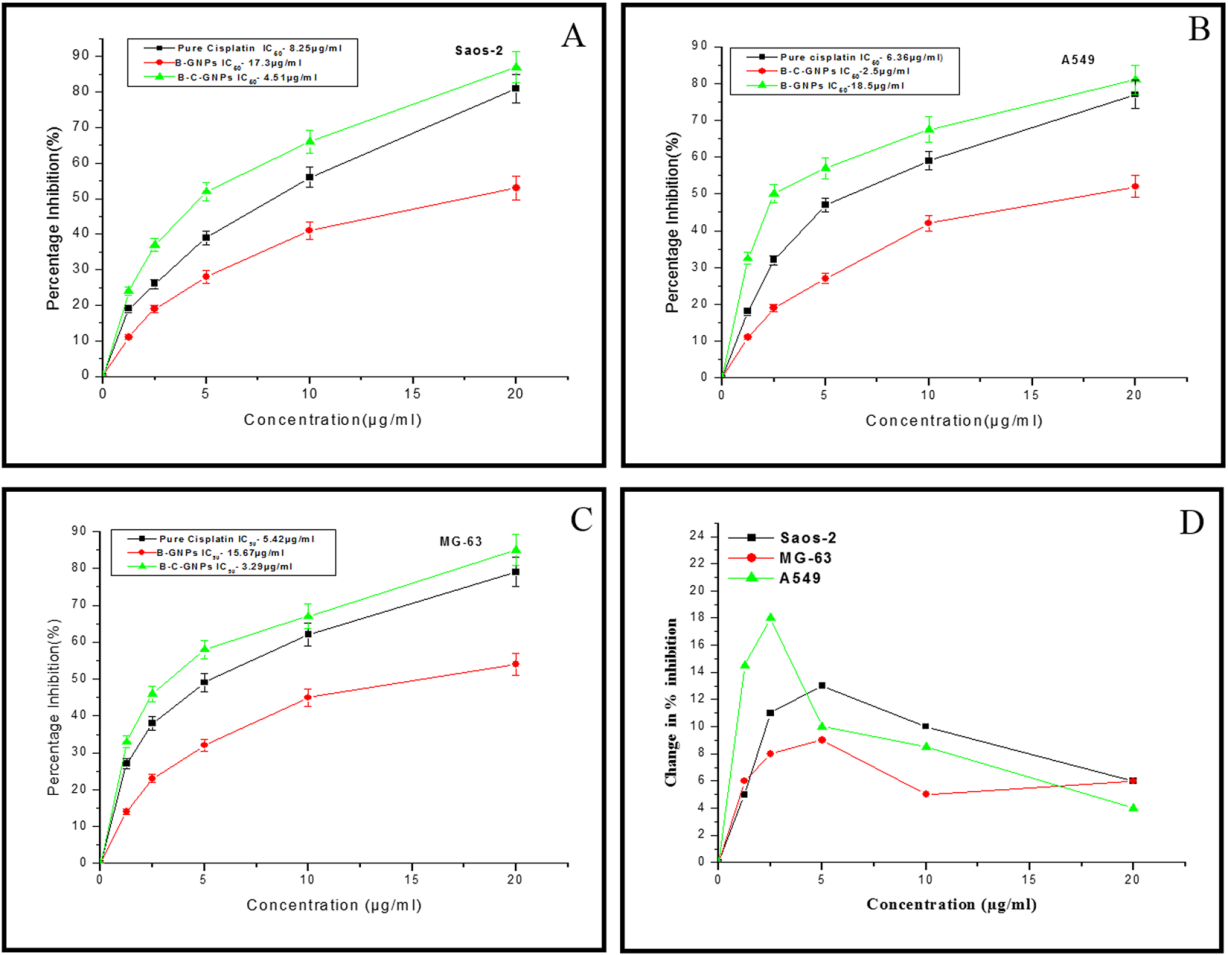
The Cytotoxicity (dose dependent) study of B-GNPs, B-C-GNPs and pure cisplatin on (**A**) Saos-2 (**B**) A549 (**C**) MG-63 cell lines and (**D**) Graph showing change in percentage inhibition between B-C-GNPs and pure cisplatin against concentration for Saos-2, A549 and MG-63. B-C-GNPs inhibited cell growth significantly of Saos-2, MG-63 and A549 with IC_50_ values 4.51 µg/ml, 3.2 µg/ml and 2.5 µg/ml, respectively and IC_50_ values of pure cisplatin on Saos-2, MG-63 and A549 were found to be 8.25 µg/ml, 5.42 µg/ml and 6.36, respectively while B-GNPs had also shown cytotoxic activity on Saos-2, MG-63 and A549 but with IC_50_ values of 17.3 µg/ml, 15.67 µg/ml and 18.5 µg/ml, respectively. All the data were expressed in mean ± SD of three experiments.

The B-C-GNPs showed a highly significant cytotoxic effect on Saos-2, MG-63 and A549 cells, which is higher than pure cisplatin drug. The percentage inhibition on Saos-2, MG-63 and A549 was discovered to be enhanced in a dose dependent manner. The IC_50_ values of cisplatin conjugated B-GNPs on MG-63, Saos-2 and A549 were found to be 3.2 µg/ml, 4.51 µg/ml, and 2.5 µg/ml, respectively which were lower than IC_50_ values of pure cisplatin on Saos-2 (Fig. 6A), A549 (Fig. 6B) and MG-63 (Fig. 6C) which were found to be 8.25 µg/ml, 6.36 µg/ml and 5.42 µg/ml respectively. B-GNPs have also shown cytotoxic activity on Saos-2, MG-63 and A549 but with IC_50_ values 17.3 µg/ml, 15.67 µg/ml and 18.5 µg/ml respectively which is much higher than allowed or permitted limit. Also, change in percent inhibition (i.e. [% inhibition of (B-C-GNPs)- % inhibition of pure cisplatin]) against concentration of respective nanomaterials used has been shown in Fig. 6D. It shows that A549 is highly affected cell line among all the tested cell lines.

### Measurement of cytomorphological changes on Saos-2

**Figure 7:**
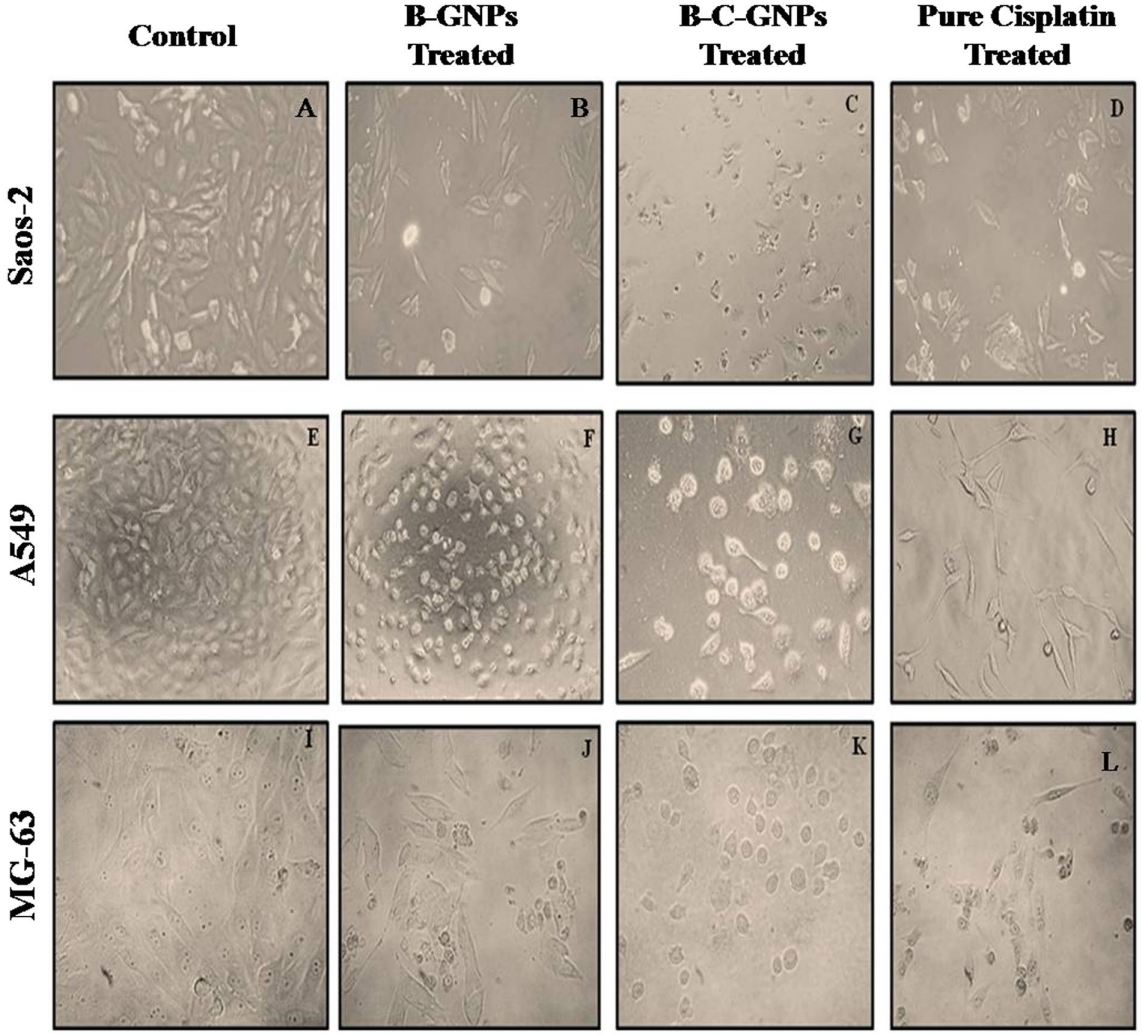
Phase contrast microscope images after 48 hrs of treatment on osteosarcoma cell lines Saos-2 & MG-63 and adenocarcinoma cell line A549 at 20X magnification. (**A**) Control of Saos-2 cells, (**B**) B-GNPs treated Saos-2 cells, (**C**) B-C-GNPs treated Saos-2 cells, (**D**) Pure cisplatin treated Saos-2 cells, (**E**) Control of A549 cells, (**F**) B-GNPs treated A549 cells, (**G**) B-C-GNPs treated A549 cells, (**H**) Pure cisplatin treated A549 cells, (**I**) Control of MG-63 cells (**J**) B-GNPs treated MG-63 cells, (**K**) B-C-GNPs treated MG-63 cells, (**L**) Pure cisplatin treated MG-63 cells.

The morphological changes in Saos-2, MG-63 and A549 cells incubated with B-GNPs, B-C-GNPs and pure cisplatin are shown under phase contrast microscopy (Fig. 7). The control cells were found to be uniformly spread and showed no distinct or remarkable changes in morphology after 48hrs of incubation (Fig. 7A,E,I). However, significant changes were observed in cells treated with B-C-GNPs (Fig. 7C,G,K) while positive control (pure cisplatin) also created considerable deformations in the cells (Fig. 7D,H,L). Treated cell, after 48 hrs of exposure, became irregular, shrinked, necrotic and detached from the surface of the wells. While some cells were found to keep their plasma membrane intact, showing that apoptosis had started. Although, pure B-GNPs also induced shrinkage and apoptosis of the cells, but at much higher concentrations as compared to B-C-GNPs (Fig. 7B,F,J).

### Analysis of changes in nuclear morphology

**Figure 8:**
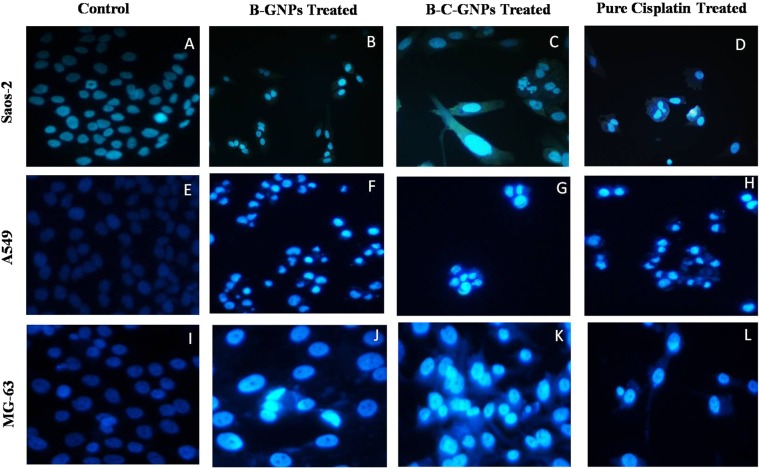
Images showing DAPI staining under phase contrast microscope after 48 hrs of treatment on osteosarcoma cell lines Saos-2 & MG-63 and adenocarcinoma cell line A549 at 20X magnification. (**A**) Control of Saos-2 cells, (**B**) B-GNPs treated Saos-2 cells, (**C**) B-C-GNPs treated Saos-2 cells, (**D**) Pure cisplatin treated Saos-2 cells, (**E**) Control of A549 cells, (**F**) B-GNPs treated A549 cells, (**G**) B-C-GNPs treated A549 cells, (**H**) Pure cisplatin treated A549 cells, (**I**) Control of MG-63 cells (J) B-GNPs treated MG-63 cells, (**K**) B-C-GNPs treated MG-63 cells, (**L**) Pure cisplatin treated MG-63 cells.

The method of cellular uptake and internalization of B-C-GNPs was additionally assessed by utilizing a fluorescent dye (4’, 6-diamidino-2-phenylindole) DAPI (Fig. 8). The B-C-GNPs treated Saos-2, A549, and MG-63 cells were incubated for 24 h at 37 °C and stained by DAPI dye. The B-C-GNPs treated cells were found to have apoptotic impact and expanded cell membrane penetrability; that brought about condensed chromatin and dark blue fluorescent consolidated nucleus (Fig. 8C,G,K) when contrasted with untreated cells (Fig. 8A,E,I). The B-GNPs also prompted apoptosis of the cells (Fig. 8B,E,J). The most critical and particular indication of cytotoxic impact caused by stress is the nucleus condensation. The positive control (pure cisplatin) likewise demonstrated noteworthy blue fluorescence in cells (Fig. 8D,H,L).

**Figure 9:**
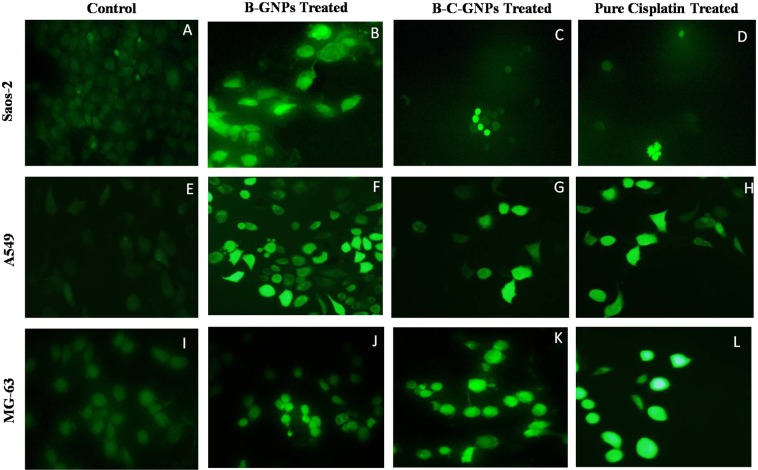
Images showing DCFDA staining under phase contrast microscope after 48 hrs of treatment on osteosarcoma cell lines Saos-2 & MG-63 and adenocarcinoma cell line A549 at 20X magnification. (**A**) Control of Saos-2 cells, (**B**) B-GNPs treated Saos-2 cells, (**C**) B-C-GNPs treated Saos-2 cells, (**D**) Pure cisplatin treated Saos-2 cells, (**E**) Control of A549 cells, (**F**) B-GNPs treated A549 cells, (**G**) B-C-GNPs treated A549 cells, (**H**) Pure cisplatin treated A549 cells, (**I**) Control of MG-63 cells (J) B-GNPs treated MG-63 cells, (**K**) B-C-GNPs treated MG-63 cells, (**L**) Pure cisplatin treated MG-63 cells.

### Estimation of reactive oxygen species

**Figure 10:**
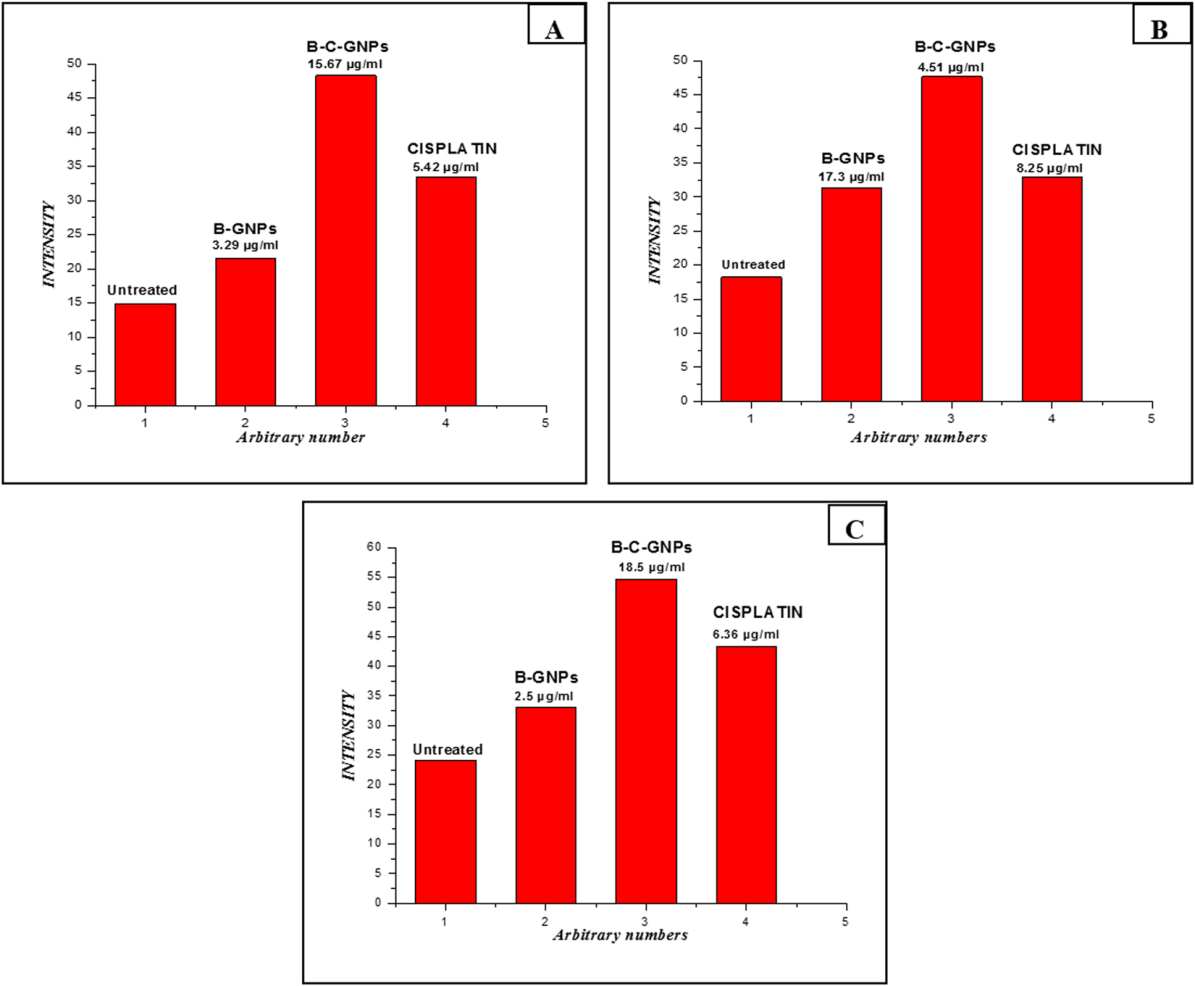
The evaluation of intensity of ROS generation in (**A**) MG-63 (**B**) Saos-2 and (**C**) A549 cells after treating with B-GNPs, B-C-GNPs and pure cisplatin at their respective IC_50_s.

The levels of intracellular ROS created in the Saos-2 cells, A549 and MG-63 cells upon interaction with B-C-GNPs, B-GNPs and pure cisplatin were estimated by utilizing 5-(and-6)- carboxy-2’,7’- dichlorodihydrofluorescein diacetate (DCFHDA) (Sigma-Aldrich) as an oxidation-sensitive fluorogenic marker of ROS in the viable cells (Fig. 9). The intensity of fluorescence was relative to the measure of ROS generated in the cells. This investigation demonstrated that B-G-CNPs treated Saos-2, A549, and MG-63 cells (Fig. 9C,G,K) indicated greater intensity of fluorescence regarding their controls (Fig. 9A,E,I). Likewise, pure cisplatin treated Saos-2, A549 and MG-63 cells (Fig. 9D,H,L) created noteworthy fluorescence and biogenic B-GNPs (Fig. 9B,F,J) emitted brilliant fluorescence with mutilated morphological structure due to aggravation disrupting impact in the compactness of plasma membrane caused by ROS produced, though unprocessed cells did not demonstrate any consideration fluorescence and held their native morphology. The cytotoxicity impacts may be applied through the generation of oxidative pressure and apoptosis with a conceivable association of overproduction of reactive oxygen species (ROS). Quantification of fluorescence using Image J software also revealed that B-C-GNPs produced more fluorescence than B-GNPs and untreated cells at their respective IC_50_s (Fig. 10).

## Discussion

Cisplatin has been widely used as a powerful therapeutic agent against numerous solid tumors, including bone and lung tumors because it is strongly potent but highly toxic^64^. Cisplatin reacts with nucleophilic centers of guanosine and adenosine residues, particularly at N7 positions. The two labile coordination sites in the platinum center permit crosslinking of adjacent guanine bases or to the lesser extent, to guanine bases from different DNA strands to form interstrand cross-links. The major intrastrand dGpG cross-link induces a significant distortion in the DNA double helix^65^. However, therapeutic applications of cisplatin have been restricted due to its nonspecific toxicity and serious side effects such as acute nephrotoxicity and chronic neurotoxicity^66^. It is chemically unstable, sparingly soluble in water, having low lipophilicity and targets healthy cells with tumor^67^. Furthermore, tumor cells may develop intrinsic resistance to cisplatin due to heavy doses^68^. In order to increase the therapeutic efficiency of cisplatin and to alleviate the limitations, Cisplatin was bioconjugated with gold nanoparticles having bromelain, a cysteine protease, as a capping agent because targeted drug delivery has advantage of low toxicity, few side effects and enhanced therapeutic efficacy. Nanoparticles with a hydrophilic surface can evade the recognition by reticuloendothelial system (RES) and bromelain is a cysteine protease and hydrophilic in nature, having several polar functional groups at its surface^69^. Also, NPs will have prolonged circulation time in the bloodstream compartment and can accumulate in solid tumors by enhancing permeation and retention (EPR) effect^70^. The bioconjugation will also block the functional groups responsible for non-specific interaction because these groups will be involved in the covalent bonding for bioconjugation between drug and NPs. Bromelain can be absorbed in human intestines without degradation and without losing its biological activity and it is well tolerated in high doses (10 g/kg) for prolonged periods of therapy^71^. The anticancerous activity of bromelain is attributed predominantly to its protease component. Bromelain is notable to enhance neutrophil movement, down regulate Cox-2, NF-κB, PGE-2^72–74^ and TGF-β^75^; upregulate Bax and p53^72^; diminish the action of cell survival regulators, for example, ErK, Akt, and deactivate Akt-dependent Pro apoptotic regulator FOXO3A^76^. It lessens articulation of CD44 on the surface of tumor cells^77^.

It has been accounted for that colloidal gold nanoparticles inside the dimension range of 3–100 nm don’t demonstrate remarkably toxicity, gave that the edge measurement does not surpass an estimation of the request of 10^12^ particles/ml^50^. The majority of proficient cellular uptake of GNPs was seen with nanomaterials extending from 20 nm to 50 nm. This bioconjugate was found to be highly effective because they lie in the best range by size and they will reduce the dose by half where side effects will also be reduced and hence, patient compliance. Additionally, cisplatin and bromelain will work synergistically as bromelain stimulates immunocytotoxicity of cancer patient-derived immune cells and initiates an intracellular cascade that negatively regulates inflammation-induced NF-κβ activation and its target gene whereas Cisplatin will bind with nucleic acid of target cells. It is assumed that bromelain will reduce the rate of formation of cancer cells while cisplatin will kill cancer cells.

## Conclusion

In this investigation, a simple process developed to synthesize gold nanoparticles using a cysteine protease bromelain. This enzyme acts in a dual mode, as reducing and capping function. These nanoparticles were conjugated with Cisplatin a well known anticancer molecule. The combination of Cisplatin bioconjugated bromelain encapsulated gold nanoparticles found potent against osteosarcoma and lung cancer at much lower concentration due to the synergy of bromelain. This nanosystem is characterized by multiple techniques that decipher its properties. The encapsulation renders the loss in secondary structure but tertiary structure was not altered and this improved anticancer potential as supported by other investigations.
